# Methods of calculating ^123^I-β-methyl-P-iodophenyl-pentadecanoic acid washout rates in triglyceride deposit cardiomyovasculopathy

**DOI:** 10.1007/s12149-022-01787-9

**Published:** 2022-09-25

**Authors:** Zhuoqing Chen, Kenichi Nakajima, Ken-ichi Hirano, Takashi Kamiya, Shohei Yoshida, Shintaro Saito, Seigo Kinuya

**Affiliations:** 1 Department of Nuclear Medicine, Kanazawa University, Kanazawa, Ishikawa Japan; 2 Department of Functional Imaging and Artificial Intelligence, Kanazawa University, Kanazawa, Ishikawa 920-8640 Japan; 3 Department of Triglyceride Science, Graduate School of Medicine, Osaka University, Suita, Osaka Japan; 4 Department of Medical Technology, Osaka University Hospital, Suita, Osaka Japan; 5 Department of Cardiology, Kanazawa University Hospital, Kanazawa, Ishikawa Japan

**Keywords:** Triglyceride deposit cardiomyovasculopathy, Fatty acid, Washout rate, Nuclear cardiology, Diagnosis

## Abstract

**Objective:**

This study aimed to optimize various methods of calculating washout rates (WRs) of ^123^I-β-methyl-p-iodophenyl-pentadecanoic (BMIPP), as they are essential to diagnose triglyceride deposit cardiomyovasculopathy (TGCV) which is a rare disease entity identified in Japan and has been encoded in Orphanet (ORPHA code 565612).

**Methods:**

We calculated WRs of ^123^I-BMIPP from early (20 min) and delayed (200 min) images. We evaluated six methods of calculating WRs to discriminate TGVC patients (age, 56.8 ± 14.6 y; male, *n* = 13; female, *n* = 4) and 21 ^123^I-BMIPP studies were involved including 4 follow-up studies. Washout rates were calculated by two planar methods using anterior images with cardiac and background regions of interest (ROIs) and by four SPECT methods using either array and polar plots or summed short-axis images. The final diagnoses of TGCV were confirmed according to the 2020 diagnostic criteria, and the diagnostic accuracy of WRs calculated using the six methods was analyzed using the area under receiver-operating characteristics curves (ROC-AUC). Multiple scatter-plot matrix methods were evaluated with correlations for comparison.

**Results:**

All six methods were useful for diagnosis and did not significantly differ. The four SPECT methods showed excellent diagnostic accuracy (AUC 1.0), whereas the planar methods with and without background correction could be acceptable (AUC 0.857 and 0.964, respectively). The WRs were relatively lower for patients with CAD and remarkable metabolic defects than for patients with TGCV but without defects.

**Conclusions:**

For the diagnosis of TGCV, the WR cutoff of 10% of ^123^I-BMIPP functioned well in planar and SPECT discrimination based on computational methods as a classifier. However, calculation optimization should improve TGCV diagnoses.

## Introduction

Triglyceride deposit cardiomyovasculopathy (TGCV) was discovered in a Japanese cardiac transplant candidate by Hirano et al. in 2008, then characterized as the massive accumulation of triglyceride in cardiomyocytes and smooth muscle cells [1, 2]. The disease is relevant to the defective intracellular lipolysis leading to excess triglyceride accumulation and loss of release of long-chain fatty acid (LCFA). The Japan TGCV study group developed the diagnostic criteria [3, 4], and classified  TGCV into the following two types: primary TGCV caused by homozygous mutations in *PNPLA2* gene encoding adipose triglyceride lipase [5] which is a vital enzyme for triglyceride metabolism [6, 7]; idiopathic TGCV, of which genetic mechanism remains unknown [8, 9]. According to the first international registry data [10], both types showed markedly reduced ^123^I-β-methyl-p-iodophenyl-pentadecanoic (BMIPP) washout rate, which reflected defective intracellular lipolysis. Patients with TGCV exhibited cardiomyocyte steatosis and diffuse concentric-type stenosis, TG-deposited smooth muscle cells in thick intima and media with synthetic markers such as SM22a, but without scavenger receptors [11]. Then, TGCV was encoded in the Orphanet (ORPHA code: 565612). Excessive TG accumulation in the myocardium and smooth muscle cells results in various complications including heart failure, cardiomyopathy, arrhythmia, coronary artery disease, chronic kidney disease, and skeletal muscle myopathy [12–14], all of which determine a poor prognosis. Early phase clinical trials with a novel orphan drug, CNT-01, have been successfully completed in Japan [15].

However, TGCV remains difficult to diagnose. This is because plasma levels of TG and body mass index are irrelevant clinical findings for TGCV [8]. With respect to this, ^123^I-BMIPP scintigraphy has been proposed as a means of assessing impaired intracellular TG and LCFA metabolism [16]. Markedly reduced ^123^I-BMIPP washout reflects defective intracellular lipolysis and subsequently favors a diagnosis of TGCV. In accordance with recent diagnostic criteria, a washout rate (WR) of < 10% in myocardial ^123^I-BMIPP single-photon emission computed tomography (SPECT) is essential for a diagnosis of TGCV [3, 17]. However, the disadvantage is that BMIPP defects in early and delayed images are bothersome, and WRs increase in patients with ischemic coronary artery disease (CAD) [18]. Various ways of calculating WRs including planar and SPECT imaging have been applied in nuclear cardiology, but whether they can diagnose TGCV has not been evaluated.

Various WR calculation methods have been proposed for nuclear cardiology based on early and delayed counts of planar and SPECT images. A visual sign in ^201^Tl myocardial perfusion scans that indicates multivessel and severe coronary artery diseases is a diffuse slow WR in circumferential profile analyses and polar maps. Abnormal regional WRs are mostly calculated from pixel or segment counts computational processes [19–21], whereas average-count-based calculations of WR are used to identify slow global washout [22, 23]. Global and regional WRs of ^99m^Tc-sestamibi have been applied to evaluate coronary artery disease, heart failure, and mitochondrial dysfunction in dilated cardiomyopathy. In addition to the above calculation methods, cardiac counts of planar anterior images, mean counts of 17-segment polar maps, and average or total myocardial counts of polar maps are also often applied to calculate WRs [24–27]. Anterior image data are frequently used in ^123^I-metaiodobenzylguanidine (MIBG) cardiological studies, whereas total myocardial voxel counts in SPECT studies might be another solution [28–30]. As an alternative to planar data, summation counts of SPECT slices have also been applied to exclude attenuation and background [31, 32]. Briefly, WRs can be calculated via various approaches depending on the purpose. However, only pixel-based WRs averaged on polar maps are being applied as a conventional method to TGCV diagnoses.

We, therefore, aimed to determine the diagnostic accuracy of six means of calculating BMIPP WRs in patients with and without TGCV and the validity of the 10% WR threshold for a diagnosis of TGCV. We also discuss pitfalls and limitations of BMIPP WRs for the diagnosis of TGCV.

## Materials and methods

### Patients

**Fig. 1:**
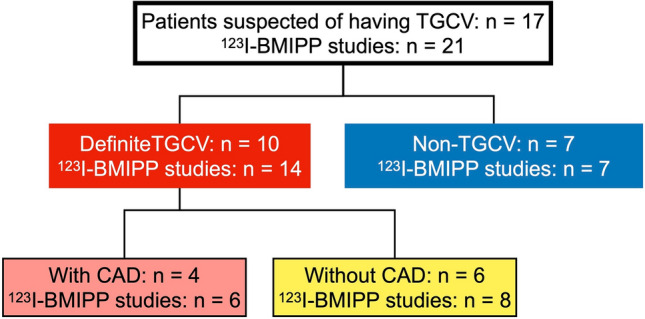
^123^I-BMIPP studies in TGCV and non-TGCV patients. TGCV, triglyceride deposit cardiomyovasculopathy

This study collected 21 ^123^I-BMIPP studies from 17 patients (age 56.8 ± 14.6 years; male, *n* = 13; female *n* = 4), and among the 21 studies, four follow-up studies from two of the 17 patients were included (Fig. 1). Intervals between the follow-up studies and the initial one were 10 and 39 months in one patient, and 44 and 93 months in another patient. We analyzed 21 image sets for subsequent analysis. Ten of 17 were diagnosed with TGCV, four of these 10 had TGCV accompanied by coronary artery disease (CAD) and seven did not have TGCV. Eleven patients were assessed by ^123^I-BMIPP SPECT at Osaka University Hospital and six were examined at Kanazawa University Hospital. None of the TGCV patients underwent stress myocardial perfusion studies when diagnosed with TGCV.

### Diagnostic criteria

Table 1 shows the current diagnostic criteria for TGCV [3]. Patients with definite TGCV in the present study met the criteria as follows. For essential items, all patients showed BMIPP WR criteria of < 10% by SPECT-based polar map analysis. Two patients showed cardiomyocyte steatosis in the endomyocardial biopsy specimens. For major items, 8, 1, and 2 patients showed the diffuse narrowing coronary artery, reduced left ventricular ejection fraction, and typical Jordans' anomaly, respectively.

**Table 1:** Diagnostic criteria for triglyceride deposit cardiomyovasculopathy (2020)

Criteria	Clinical findings
Essential	Impaired LCFA metabolism or TG deposition in myocardium
	1. Decreased washout rate (< 10%) in myocardial ^123^I-BMIPP SPECT
	2. Myocardial TG deposition by biopsy specimens
	3. Myocardial TG deposition by CT or MR spectroscopy
Major	1. Decreased left ventricular ejection fraction < 40%
	2. Diffuse narrowing of coronary arteries documented by CAG and/or coronary CT angiography
	3. Typical Jordans anomaly (apparent vacuoles > 1 μm in size) of polymorphonuclear leucocytes in peripheral blood smears
Supportive	1. Diabetes mellitus
	2. Hemodialysis

Diagnosis Definite TGCV: Meets one or more essential criteria and one or more major criteria

Probable TGCV: Meets at least one essential criterion

^123^I-BMIPP, ^123^I-β-methyl iodophenyl-pentadecanoic acid; *CAG* coronary angiography, *CT* computed tomography, *LCFA* long chain fatty acid, *MR* magnetic resonance, *SPECT* single-photon emission computed tomography, *TG* triglyceride, *TGCV* triglyceride deposit cardiomyovasculopathy

### ^123^I-BMIPP SPECT/CT

Patients underwent ^123^I-BMIPP scintigraphy at 20 and 200 min after intravenous injection of 111 MBq (3 mCi) of ^123^I-BMIPP (Nihon Medi Physics, Co. Ltd., Tokyo, Japan) for early image acquisition and delayed data collection, respectively.

The BMIPP protocol at Osaka University Hospital was as follows. We acquired anterior planar images for 5 min before starting the SPECT study for early and delayed images collection respectively, using a dual-head gamma camera (BrightView; Philips Healthcare, Cleveland, OH, USA) with a cardiac high-resolution collimator. The SPECT images were acquired in step-and-shoot mode with 64 projections collected for 40 s per view, and the pixel size was 6.4 mm. The energy discrimination was centered at 159 keV with a 20% window. Short, vertical long, and horizontal long axes images were created with a 64 × 64 matrix using ordered subset-expectation maximization (OSEM). The order of the Butterworth filter was 5 and the cutoff frequency was 0.39 cycles/cm.

A triple-head gamma camera (GCA-9300R; Canon Medical Systems Corp., Otawara City, Japan) equipped with a low-medium energy general-purpose collimator was rotated in a 360° arc in a circular orbit at Kanazawa University Hospital. The energy discriminator of the camera was set at the 159-keV photopeak of ^123^I with a 20% window. Data were collected in a 128 × 128 matrix with 3.2-mm pixels. Raw imaging data were reconstructed using a Butterworth filter (order, 4; cut-off frequency, 0.40 cycles/cm and OSEM. Short- and long-axis tomograms were computed and displayed.

The interval between two consecutive R waves of the electrocardiogram (RR) was divided into 16 frames to create gated images. We obtained 60 projection images for 60 s per step over 360°. Tracer uptake was assessed using non-gated images, and summed images were created from all gated images acquired in standard mode.

### Ethics approval

The Ethics Committees of Kanazawa University and Osaka University approved the protocol of this retrospective study. Written informed consent was waived due to the retrospective collection of the clinical data; however, all patients had the right to opt-out of the study at any time.

### Data processing

All data in Digital Imaging and Communication in Medicine (DICOM) format were processed using Mathematica (v. 12.3.1.0, Wolfram Research, Inc., Champaign, IL, USA). Washout rates were calculated with time decay corrections in both planar and SPECT methods; i. e. the decay correction factor was 1/0.5^(time/13.2 h) for delayed images. A background (BG) subtraction was only performed on planar images, as SPECT data do not include BG counts.

### Washout rate calculation based on planar images

All imported images were resized to 256 × 256 pixels in grayscale. A circular region of interest (ROI) was placed over the entire heart and a rectangular ROI over the upper mediastinum was the reference BG region in the planar anterior images. Both early and delayed images were adjusted to 128 × 128 pixels for calculations. The heart to mediastinum (H/M) ratio and the global WR of BMIPP were calculated from the counts in ROI after time decay corrections as follows:

H/M ratio (HMR) = $$\frac{\mathrm{mean \, cardiac \, count }}{\mathrm{mean \, mediastinal \, count}}$$

Washout rate without background correction (Method PL_n_) (%) = $\frac{\left(\mathrm{MECC}-\mathrm{ MDCC}\right)}{\mathrm{MECC}}\times 100$

Washout rate with background correction (Method PL_b_) (%) = $\frac{\left(\mathrm{MECC}-\mathrm{ MEMC}\right)-(\mathrm{MDCC}-\mathrm{MDMC})}{\mathrm{MECC}-\mathrm{ MEMC}}\times 100$

MECC, mean early cardiac count; MEMC, mean early mediastinum count; MDCC, mean delayed cardiac count; MDMC, mean delayed mediastinum count.

### Washout rate calculation based on SPECT images

Short-axis SPECT data were imported in DICOM format and slices that did not include the myocardium were excluded. Early and delayed DICOM images were imported to create short-axis sum (SAsum) images and adjusted for the time-decay correction. The most apical and basal slices that included extracardiac activity were excluded; that is, only ring-shaped slices were selected to avoid partial volume effects at both ends. The total counts on the early and delayed SAsum image were used for calculating WRs.

Myocardial regions were selected by appropriate zooming, and early and delayed image locations were co-registered to create polar maps. We created 120 sectors × 20 slices of array data using the standard algorithm for calculating polar maps and HRV-S software (Nihon Medi Physics, Tokyo, Japan). Pixel-based or sector WRs were calculated after time decay correction based on the array data.

Basically, the WR (%) was calculated as:

$$\frac{\mathrm{Early cardiac counts}-\mathrm{Delayed cardiac counts}}{\mathrm{Early cardiac counts}}\times 100$$

**Fig. 2:**
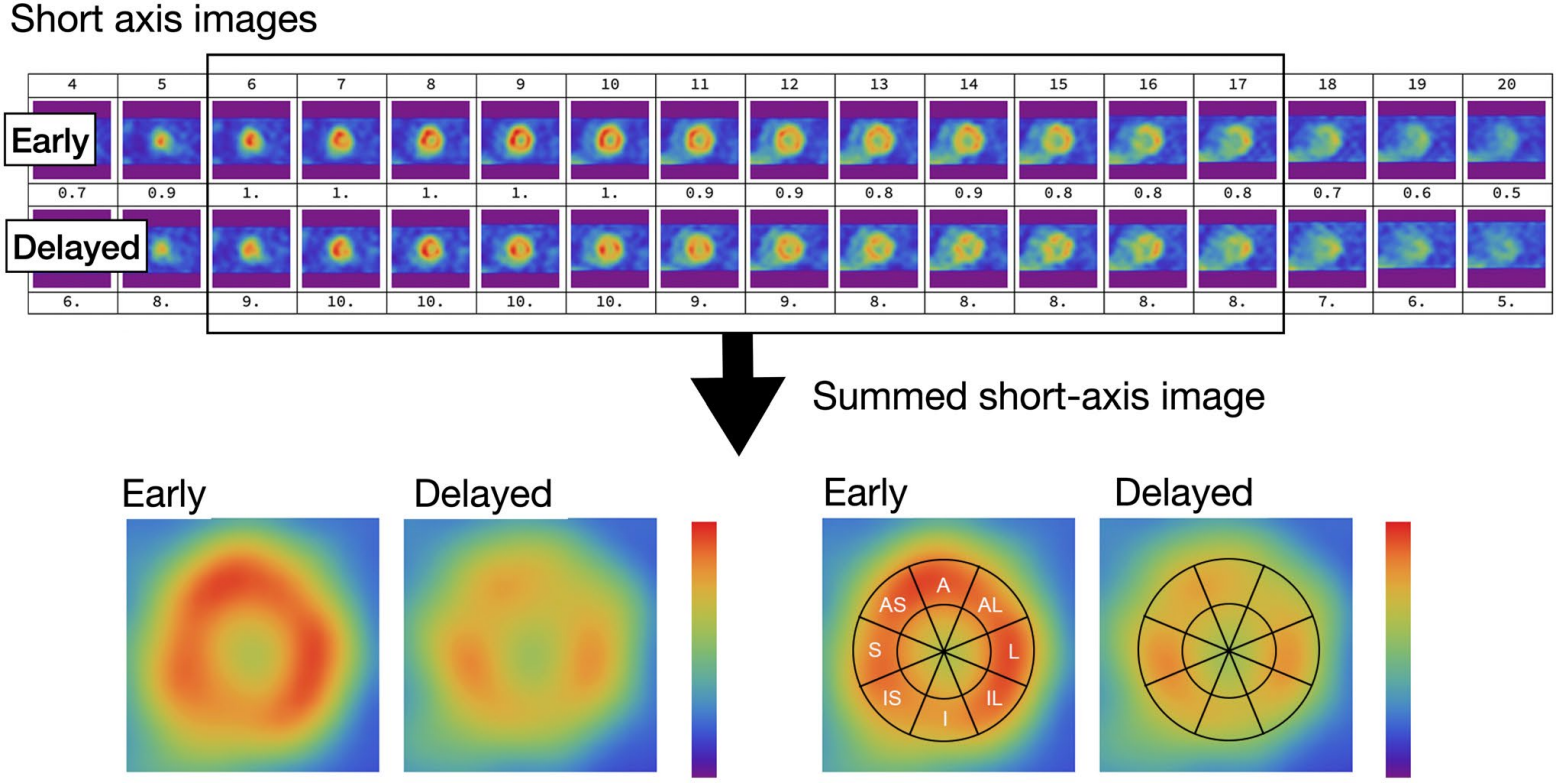
Calculations based on SPECT summed images. *A* anterior, *AL* anterior lateral, *AS* anterior septum, *Av* average, *I* inferior, IL, inferior lateral; IS, inferior septum; S, septum

Early and delayed short-axis slices were fused into a summed image, then the following calculation methods were applied (Fig. 2). Total counts of SAsum images were taken as cardiac counts in Method S_sum_, and summed images were equally divided into eight sectors, the WR of each was calculated and averaged, and counts in the left ventricular cavity were excluded from Method S_sec_.

**Fig. 3:**
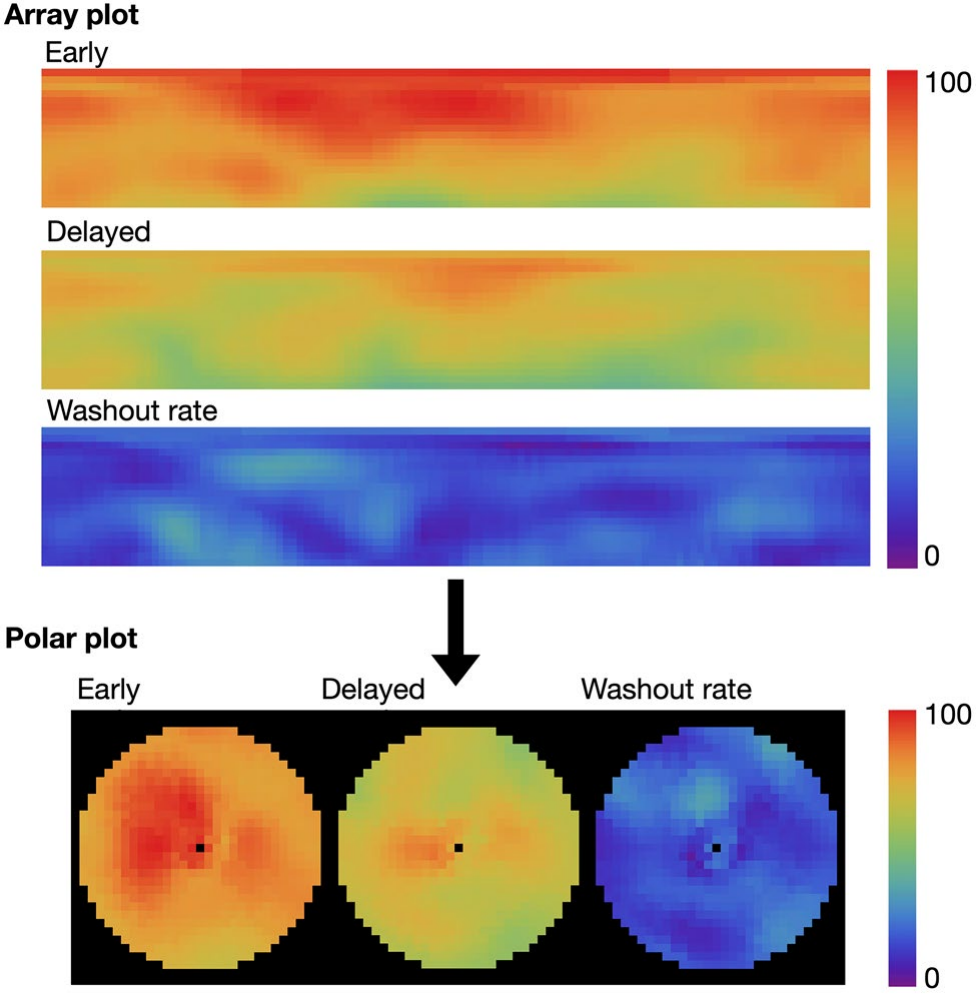
Calculations based on array plots and polar maps. Images were derived from processed data from a patient with TGCV

In addition to the above, the array data used to create polar maps with the short axis from the apical to the basal slices and the two calculation methods were applied as follows (Fig. 3).

In Method PM_ave_, averaged counts of polar maps were taken as cardiac counts and averaged counts in the two most basal and apical rows were excluded to avoid extracardiac activity involvement and mismatches in the selection of border slices. In Method PM_px_, the WRs were individually calculated for all pixels and averaged. The WR was similar to the output results of the HRV-S software except for excluding the most basal and apical slices.

### Reproducibility of washout rate

Three representative BMIPP scintigraphy scans were selected to evaluate WR variations when using the same method, and the three datasets chosen were collected from a patient with TGCV but without CAD, a patient without TGCV nor CAD, a patient with TGCV and CAD, and a patient with BMIPP metabolic defects caused by CAD but not diagnosed with TGCV.

Every calculation method of the six was reperformed to the three patients five times. For planar-image-based methods, each time when computing, the sizes and the positions of ROIs were intentionally adjusted within a proper range. In the two polar-map-based methods, when generating the polar maps, the range of selected slices from the base to the apex was intentionally changed by several slices. For the other two SAsum methods, the selection range of slices and the numbers of slices chosen to process were altered within technically plausible variation.

### Statistical analyses

All results are shown as means ± standard deviation (SD) or standard error. The significance of differences between groups was assessed by one-way analysis of variance (ANOVA) and *t* tests. The diagnostic metrics of accuracy, sensitivity, and specificity at a diagnostic cutoff of WR 10%, and the area under the receiver-operating characteristic (ROC) curve (AUC) were calculated. Multiple regression fit models between methods were evaluated with correlations for comparison. A P value < 0.05 was considered statistically significant. All the statistical analyses were performed by JMP Pro software (version 16.0.0, SAS Institute, Cary, NC, USA).

## Results

### Characteristics of the patients

The average age of the patients was 56.8 ± 14.6 years (male *n* = 13; 76%), and 8 (47%) had CAD. ^123^I-BMIPP SPECT images revealed large BMIPP defects in six patients, and four of 17 patients were diagnosed with TGCV accompanied by CAD.

### Reproducibility of washout rate

All four SPECT methods showed satisfactory reproducibility of WR within ± 1.5% (SD), while Method PL_n_ and Method PL_b_ exhibited relatively wider variations (SD 5% and 11%, respectively) as listed in Table 2.

**Table 2:** Reproducibility of washout rate calculations

Case		PL_b_	PL_n_	PM_px_	PM_ave_	S_sum_	S_sec_
TGCV without OMI							
Mean (%)		− 7.2	2.0	− 1.8	− 1.8	− 1.5	0.0
SD (%)		3.2	1.6	2.5	2.1	2.3	0.2
Non-TGCV without OMI							
Mean (%)		26.4	11.6	18.0	17.2	17.2	21.2
SD (%)		7.4	3.2	0.7	0.6	1.9	1.3
TGCV with large metabolic defects							
Mean (%)		1.6	− 0.8	− 1.0	0.0	− 4.2	0.4
SD (%)		10.0	2.4	1.4	1.5	1.1	1.3
Non-TGCV with defect caused by OMI							
Mean (%)		21.4	4.0	− 8.2	5.5	3.6	11.8
SD (%)		11.2	4.7	1.5	1.2	1.5	1.1

Six methods are as follows: PL_b_, planar method with background correction; PL_n_, planar method without background correction; PM_px_, polar map method with pixel-based calculation; PM_ave_, polar map method with average count; S_sum_, short-axis sum (SAsum) image method with total count calculation; S_sec_, SAsum method with eight-sector calculation, *OMI* old myocardial infarction, *TGCV* triglyceride deposit cardiomyovasculopathy

### Washout rates based on planar images

**Fig. 4:**
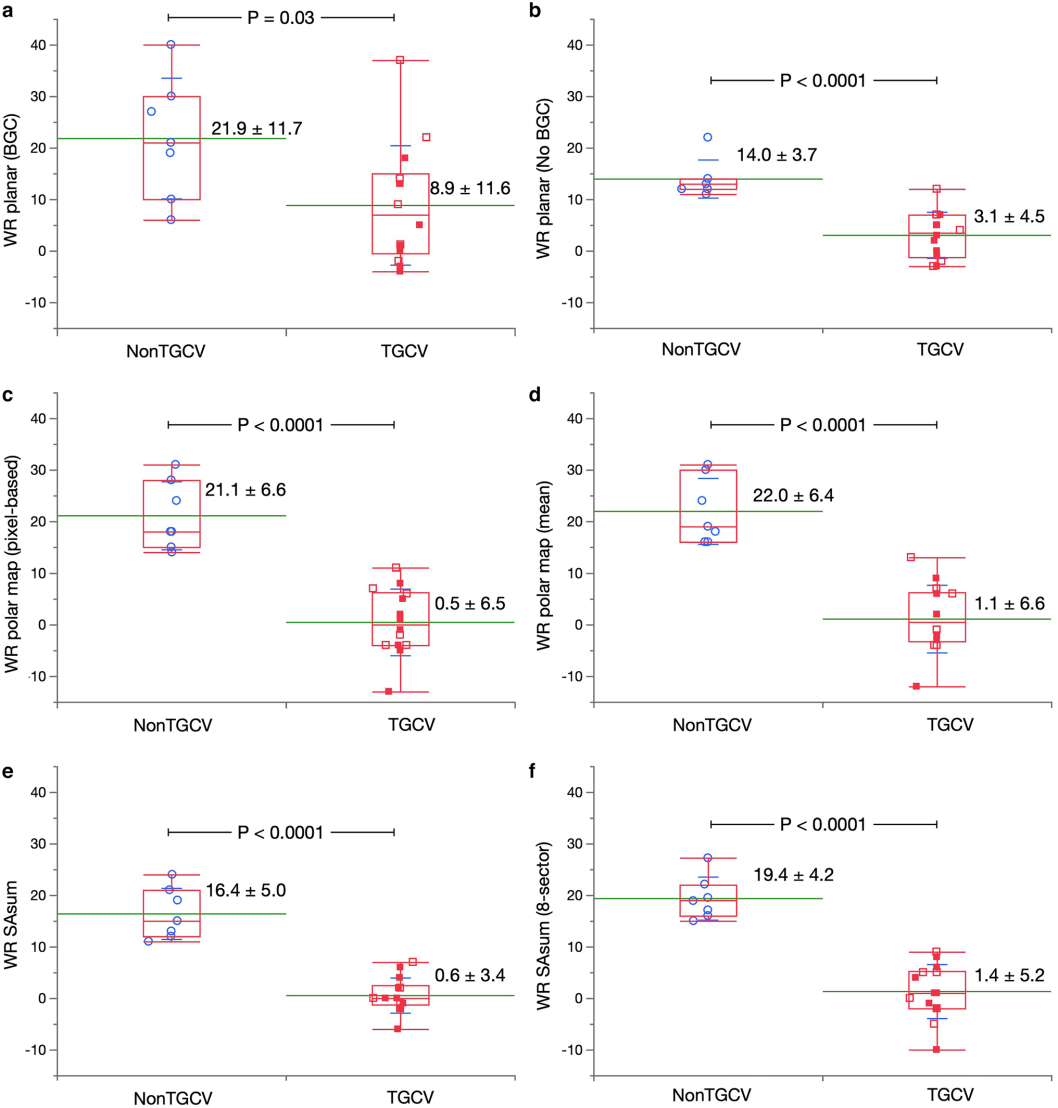
Washout rates (%) based on SPECT images from patients with and without TGCV. a–f, Methods PL_b_, PL_n_, PM_px_, PM_ave_, S_sum_, and S_sec_, respectively. Blue circle, red open and solid squares, respectively indicate non-TGCV, TGCV with and without extensive metabolic defects due to coronary artery diseases. The box plot denotes median and first and third quartiles and whiskers for value ranges. BGC, background correction; HRVS, Heart Risk View-S; SA, short-axis; seg, segment; TGCV, triglyceride deposit cardiomyovasculopathy; WR, washout rate

The WRs computed with (Fig. 4a) and without (Fig. 4b) BG correction significantly differed between the patients with and without TGCV (8.9 ± 11.6% *vs.* 21.9 ± 11.7%, *P* = 0.03, and 3.1 ± 4.5% *vs.* 14.0 ± 3.7%, *P* < 0.0001), respectively.

### WR based on SPECT images

The BMIPP results revealed notably decreased WRs in the patients who are with TGCV, compared with those who are not (*P* < 0.0001). Figure 4c, d, e, f shows the specific outcomes of ANOVA and Student *t* tests.

### Diagnostic efficiency

Planar-images-based calculation: Diagnostic efficiency was relatively better for the method without, than with BG correction (Table 3).

**Table 3:** Diagnostic analyses of all the washout rate calculation methods

Method	*R*^2^	*P*	AUC	Cutoff (%)	Specificity	Sensitivity
PL_b_	0.25	0.0237	0.857	10.0	0.71	0.75
PL_n_	0.74	< 0.0001	0.964	10.0	0.86	0.88
PM_px_	1.00	< 0.0001	1.000	10.0	1.00	0.90
PM_ave_	1.00	< 0.0001	1.000	10.0	1.00	0.90
S_sum_	1.00	< 0.0001	1.000	10.0	1.00	1.00
S_sec_	1.00	< 0.0001	1.000	10.0	1.00	1.00

*PL*_*b*_ planar method with background correction, *PL*_*n*_ planar method without background correction, *PM*_*px*_ polar map method with pixel-based calculation, *PM*_*ave*_ polar map method with average count, *S*_*sum*_ short-axis sum (SAsum) image method with total count calculation, *S*_*sec*_ SAsum method with eight-sector calculation

Calculations based on SPECT images: The diagnostic performance of all four computational methods was remarkable (AUC = 1.000) (Table 3). Though all 4 SPECT methods showed superior AUC values, the two methods using polar maps presented a sensitivity of 0.90 at the cutoff value of 10.0%.

### Comparison of six methods

**Fig. 5:**
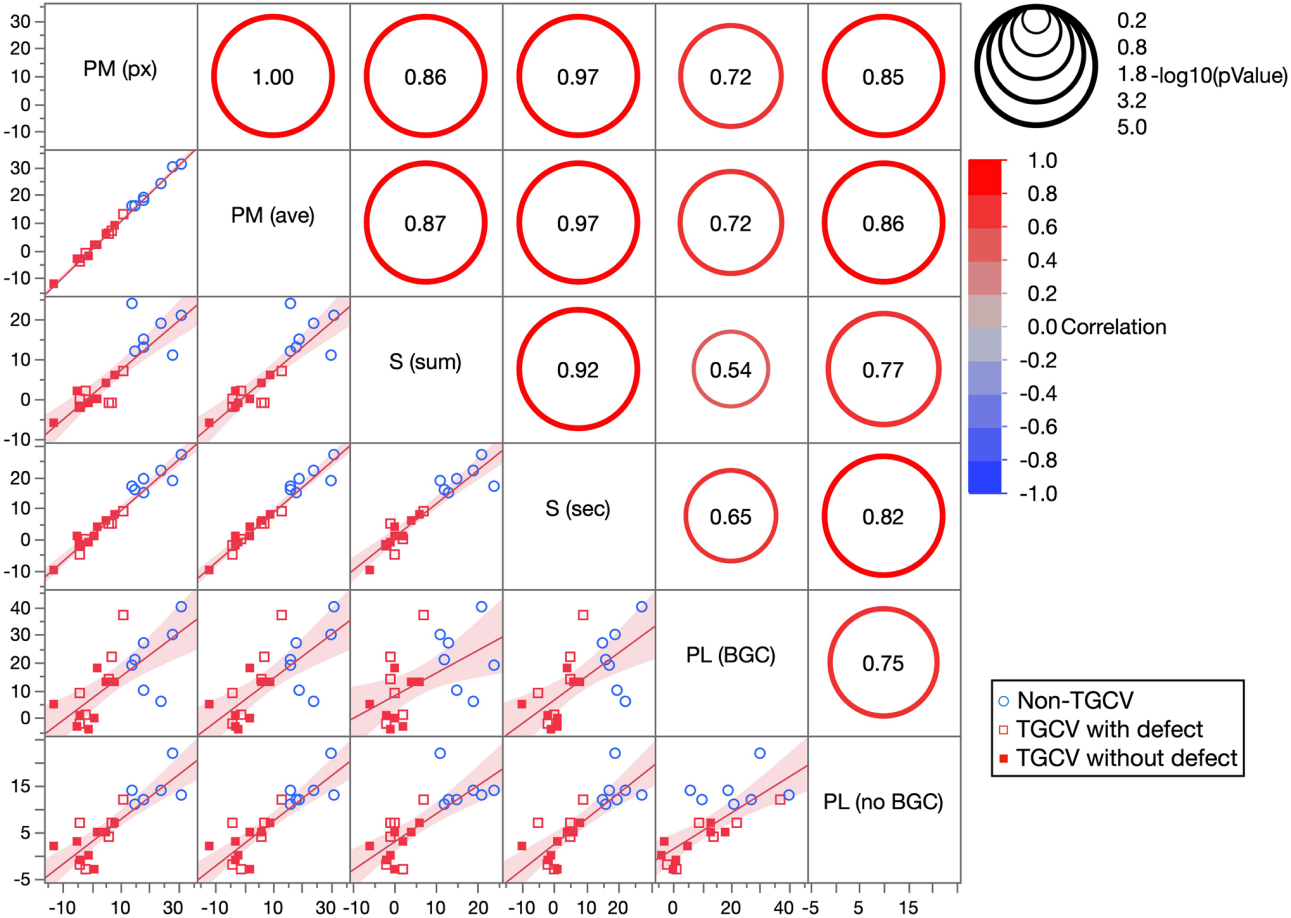
Scatterplot matrix of six methods for calculating washout rates. Correlations among Methods PM_px_, PM_ave_, S_sum_, S_sec_, PL_b_, and PL_n_, were estimated. Correlations are displayed in sizes of circles, and circle scale for reference is on the right side of graph. Blue circle and red open and solid squares, respectively, indicate non-TGCV, TGCV with and without extensive metabolic defects

**Fig. 6:**
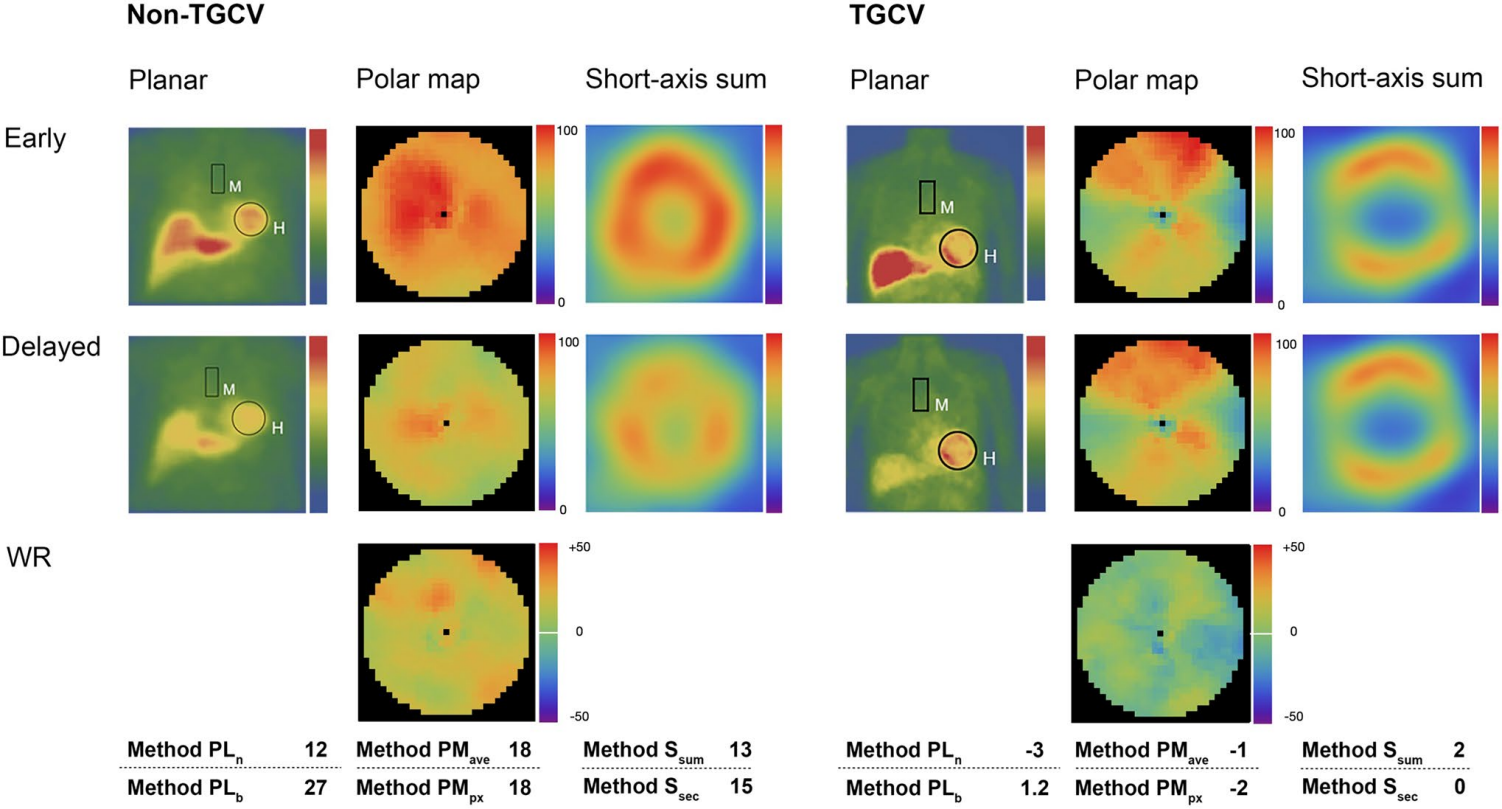
Comparison of six methods between patients with and without TGCV. Color scales of all delayed planar images, polar maps, and short-axis sum images were standardized to the maximum count of early images after time-decay correction. Myocardial counts are decreased and similar (same color) in delayed, compared with early images in patients without and with TGCV, respectively.

The scatterplot matrix in Fig. 5 shows that both methods based on planar images were less stable than those based on SPECT images. All correlations are listed in the matrix graph. Two typical patients are shown with ^123^I-BMIPP images and WR results by six methods (Fig. 6). All the WRs were > 10% in the non-TGCV patient, whereas they were < 10% in the TGCV patient. Moreover, the follow-up BMIPP studies (*n* = 4) of the two patients showed that the WRs continued to be less than 10% irrespective of six WR calculation methods, and they did not have any acute coronary events during follow-up.

## Discussion

The BMIPP WRs calculated from planar data sets were initially employed to diagnose TGCV. However, the four methods based on SPECT images diagnosed TGCV more efficiently than those using planar images when the cutoff was 10%, which further supported the vital role of BMIPP scintigraphy for diagnosing TGCV.

### Planar-image-based calculation methods

The diagnostic performance of the methods based on planar images was relatively unsatisfactory. This might have been attributed to the excessive deviation of the WRs, which were worse with BG correction and rendered classification even more troublesome. Additionally, the computational process of both calculations included chamber counts, which cannot be avoided in the planar method. The diagnostic efficiency of the method without BG correction was sufficient (*R*^2^ = 0.74, *P* < 0.0001, AUC = 0.964, sensitivity = 0.88, specificity = 0.86); indeed, calculation errors due to severe myocardial ischemia defects remained and affected the results. Thus, although planar methods can be used for the diagnosis of TGCV as supporting data, we recommend the method without BG subtraction considering the better reproducibility than that with BG subtraction (Table 2).

### Comparison with conventional method

Table 4 shows the characteristics of each method. The conventional calculation method in clinical practice (namely, pixel-based WR averaged on polar maps) and the Method PM_px_ herein are basically the same except for the exclusion of most basal and apical slices. Calculation ranges especially on apical and basal slices are normally set by nuclear medicine technicians during clinical practice. However, these can affect WRs, which leads to some error in the conventional method that calculates WRs from per pixel counts. Compared with the conventional method and the other approaches using short-axis tomographic images, methods using summed images such as method S_sec_, and planar images without BG correction, can reduce the impact of defects caused by myocardial ischemia to some extent. Considering the influence of artifacts in the apical and the basal slices that might account for errors when calculating WR, these slices were correctly omitted even for Methods S_sum_ or S_sec_. Notably, the conventional polar map method usually excludes cardiac chamber counts whereas the Method S_sum_ includes intra-cavitary counts like methods based on planar images.

**Table 4:** Comparison of methods to calculate washout rates

Method	PL_n_	PL_b_	PM_px_	PM_ave_	S_sum_	S_sec_
Definition	Planar without BG correction	Planar with BG correction	Polar map Pixel-based WR	Polar map Averaged counts	SAsum total	SAsum 8 sectors
Planar/SPECT	Anterior image	Anterior image	SPECT	SPECT	SPECT short-axis image	SPECT short-axis image
Averaged myocardial wall counts	Yes	Yes	–	–	Yes	Yes
Max count on profile curve	–	–	Yes	Yes	–	–
Background exclusion	No	Yes	Yes	Yes	Yes	Yes
Defect exclusion	No	No	Possible	No	No	Possible
Cavity count exclusion	No	No	Yes	Yes	No	Yes
Overlapping organ count	Yes	Yes	No	No	No	No

Abbreviations of six methods: see Table 2 footnotes

The approach using summed short-axis images to calculate total myocardial counts has been applied to calculate the right-to-left myocardial uptake ratio for estimating right ventricular pressure overload and ^123^I-MIBG heart-to-mediastinum count ratios with IQ-SPECT [31, 32]. Maximum counts on profile curves were used to create polar maps, and pixel-by-pixel washout was averaged for analysis. Defective regions and mismatched slices significantly influenced average counts especially when selected slices shifted. In contrast, summed images include the total myocardial wall count, and the total count is not susceptible to myocardial wall thickness, noise, and regional defects that are relatively small, which makes the WR relatively stable compared with averaged per-pixel analysis. In addition, short-axis images can be summed in any dedicated nuclear medicine processing software, which is an advantage of this method. However, the inclusion of cavitary counts might be a weakness. Therefore, we analyzed eight sectors to avoid the effects of the cavitary activity. Nonetheless, increased cavitary counts in delayed images might cause errors in WR calculation, and the PM_ave_, PM_px_ and S_sec_ methods could be used. Lastly, when myocardial defective regions had outlier regional WRs, defective segments were eliminated by the Methods PM_px_ and S_sec_.

### Pitfalls of BMIPP washout rates

The following pitfalls of BMIPP WRs are known.

Pseudonormalization, in other words, a paradoxical increase of BMIPP WRs, has been reported in patients with TGCV who suffer from coronary events [15], even though its mechanism remains to be clarified. In fact, the WRs of 3 patients (6 studies) in the present study were relatively high (Fig. 4; hollow squares).

**Fig. 7:**
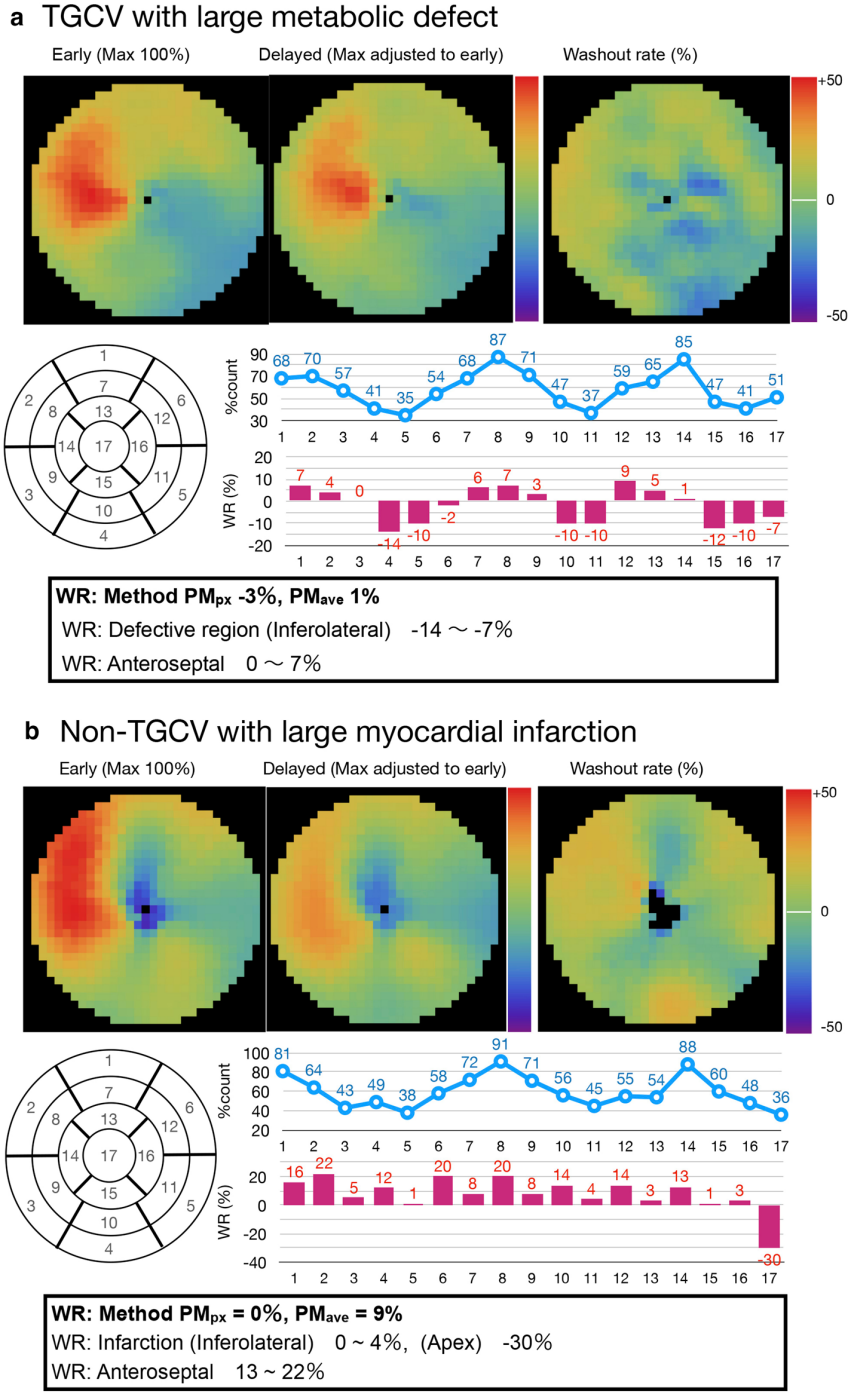
TGCV and non-TGCV patients with large metabolic defects. Segmental counts (%) on the early map (blue line graph) and WR (magenta bar graph) are shown for comparison

In addition, it should be noted that the underestimation of BMIPP WRs could occur in non-TGCV patients who mimic TGCV, as shown in Fig. 7. The upper panel displays a male patient (age, 30’s) with TGCV who has defects in both early and delayed polar maps, while the lower panel exhibits a male patient (age, 70’s) without TGCV but diagnosed with old myocardial infarction (OMI) that causes BMIPP metabolism deficiency. BMIPP scintigraphy of the non-TGCV patient in the figure was performed separately from this study. In the patient with TGCV, the mean segmental WR value (Method PM_px_) was -3% and the global average WR value (Method PM_ave_) was 1%. In contrast, the OMI patient without TGCV has a WR of 0% using Method PM_px_, but a WR of 9% using Method PM_ave_ which is borderline for TGCV diagnosis. All the global WR results were reduced due to the extremely low WRs in the inferolateral regions in both cases, which illuminates that both global and regional WRs are important in patients with a large metabolic defect. For example, the regions where metabolism was preserved (e.g., > 50%) in the TGCV patient showed segmental WR of 17 segments at 0–7% while in the non-TGCV patient the segmental WR was 13–22%, so the latter OMI patient was differentiated from TGCV. Since the pattern of defects in patients with CAD varies, regional distribution besides global metabolism is worth paying attention to for a proper diagnosis.

To resolve the above issues, integrated judgment is required taking both global and segmental WRs together with clinical history and other laboratory modalities into consideration. Table 3 shows that the 10% cutoff for diagnosis was sufficient and required no adjustment, though patients on the borderline of 10% WR exist. It is undeniable that a slight shift of the 10% threshold could occur depending on WR calculation methods, especially in planar studies. Multiple calculation procedures may, therefore, help TGCV diagnoses when encountering borderline WRs.

### Limitations

Since the discovery of TGCV in 2008, only more than 200 TGCV patients has been identified by the Japan TGCV study group [17]. Hence the small patient cohort could account for the limitations of the statistical analyses. Furthermore, raw data for this study were collected from two facilities where the acquisition conditions might have subtle differences. Considering that the WR calculation is based on the ratio of early and delayed images (or essentially the same early and delayed cardiac counts in TGCV), system error caused by scanning conditions were significantly reduced but remains indisputable. Although relatively few patients have been diagnosed with TGCV, the potential number of TGCV patients is estimated to be 1 to 2500–3000 in the Japanese general populations [18]. Further studies should include continued improvement and optimization of the algorithm for image processing to achieve an earlier and more accurate diagnosis, and the latest launched cadmium-zinc-telluride camera should be involved.

## Conclusions

^123^I-BMIPP scintigraphy is vital for TGCV diagnosis, and various approaches for calculating WRs and the 10% cutoff function well for a diagnosis of TGCV. Although planar-images-based methods, especially that with BG correction, are relatively unstable for classification, all six methods could diagnose TGCV. Further studies should promote the diagnosis of TGCV worldwide.
